# Supplementary material for: Single-cell allele-specific expression analysis reveals dynamic and cell-type-specific regulatory effects
Source: Nat Commun. 2023 Oct 9;14:6317. doi: 10.1038/s41467-023-42016-9 (PMC10562474; doi:10.1038/s41467-023-42016-9)
Supplement: Supplementary file 1 — Supplementary Information [file 41467_2023_42016_MOESM1_ESM.pdf]

Supplementary Figures

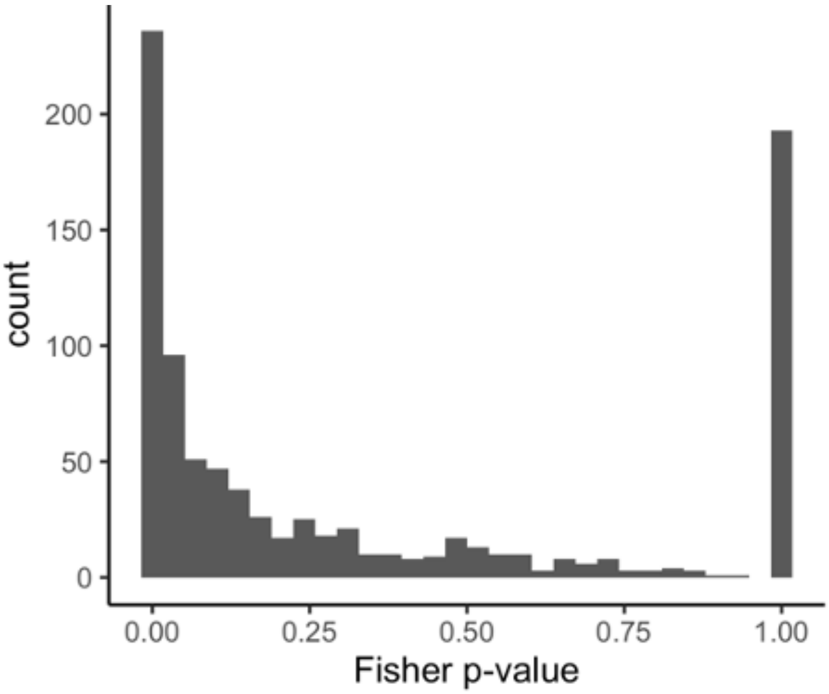

Example: p-value = 1.27e-7
Fisher's exact test

	$z_{true} = -1$	$z_{true} = 0$	$z_{true} = 1$
$z_{est} = -1$	9	18	1
$z_{est} = 1$	1	26	30

Supplementary Figure 1. Accuracy of implicit haplotype phasing by DAESC-Mix in simulation studies. Allele-specific read counts are simulated from beta-binomial mixture model for assuming only one eQTL drives ASE at a tSNP. Simulated data consist of 4,000 genes with varying LD between the eQTL SNP and the tSNP: $r^2 = 0, 0.1, 0.5, 0.9, 1$ (800 genes each, 400 null and 400 non-null). See **Methods** for details of the simulation settings. Implicit phasing is conducted for differential ASE analysis along a continuous cell state. We test the association between the true phase (z_{true}) and estimated phase (z_{est}) using a contingency table as shown above and Fisher's exact test. Only genes with at least two true phases and two observe phases are tested.

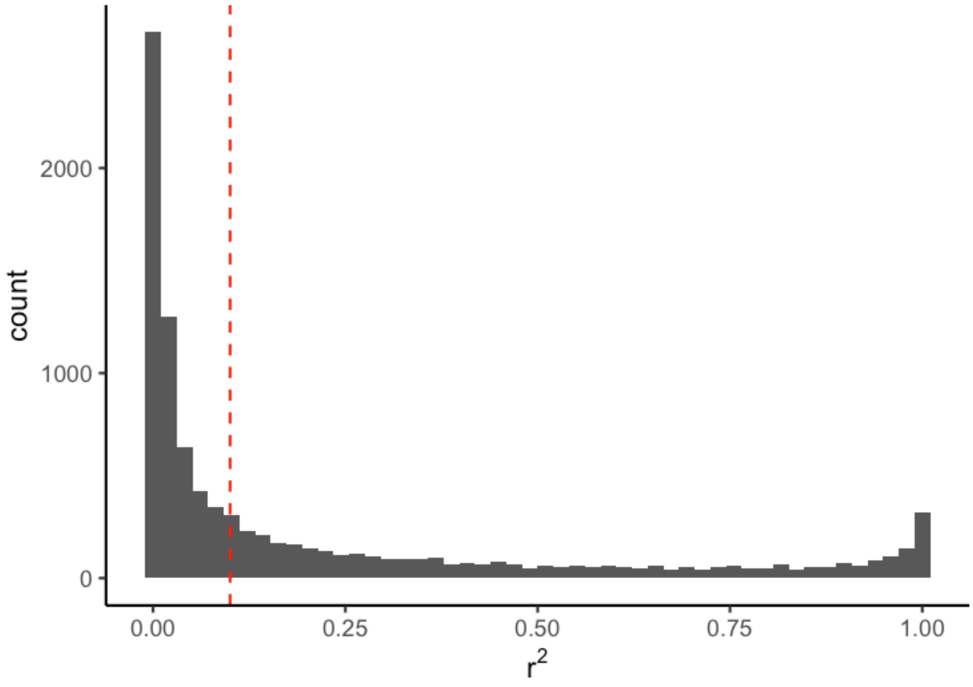

Supplementary Figure 2. Distribution of linkage disequilibrium (LD) coefficient between the top eQTL and top transcribed SNP (tSNP) of an eGene observed in GTEx v8 whole blood. The top tSNP is defined as the tSNP with highest allele-specific read depth. The top eQTL is defined as the one with smallest association p-value with the expression of the gene. The red dashed line is r^2=0.1 and 56.7% eQTL-tSNP pairs have r^2<0.1.

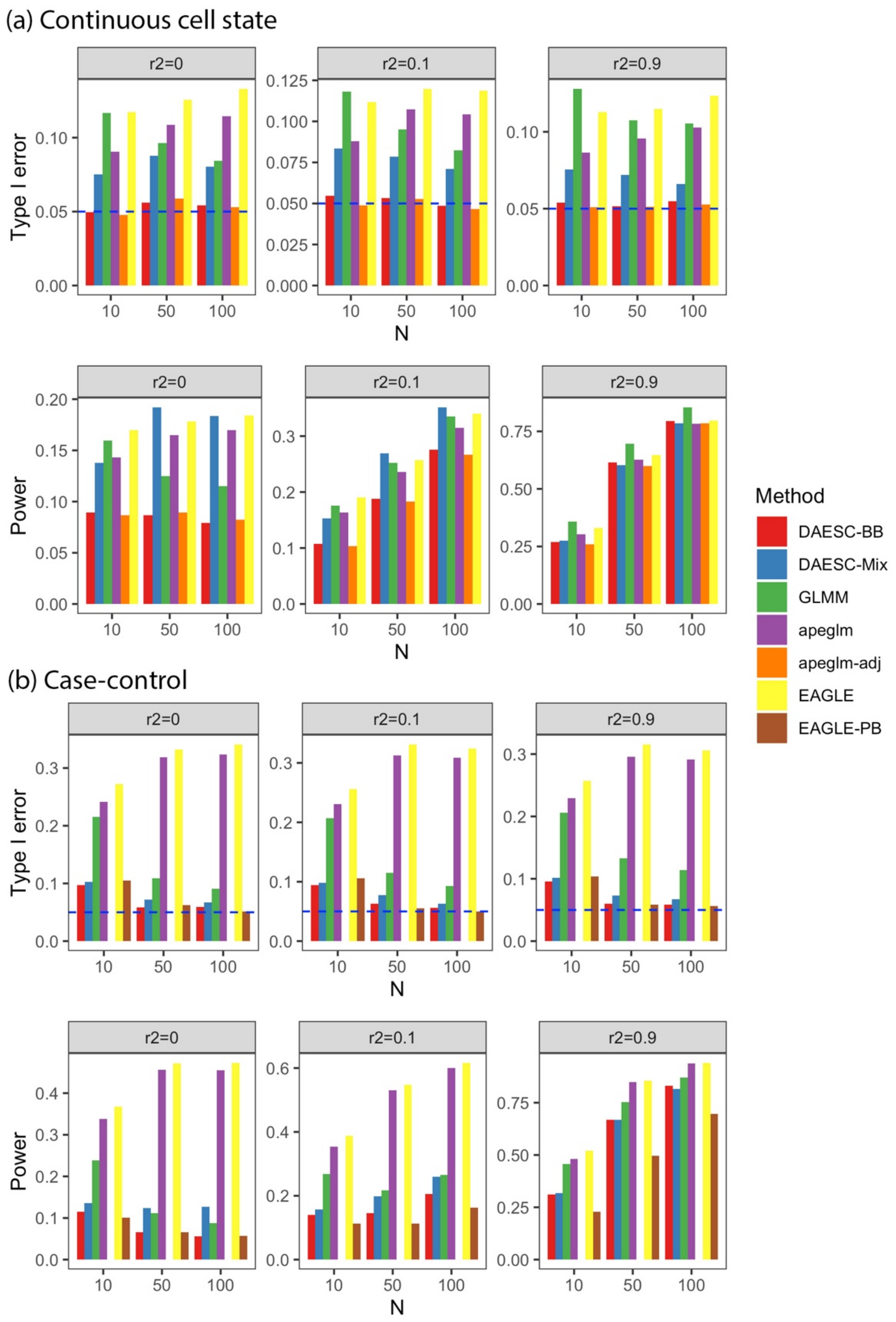

Supplementary Figure 3. Type I error and power for all methods compared in simulation studies. Allele-specific read counts are simulated from beta-binomial mixture model under the scenario where only one eQTL drives the ASE of a transcribed SNP (tSNP, see Methods). The linkage disequilibrium between the eQTL and the tSNP is varied to $r^2 = 0, 0.1, 0.9$, and the sample size is varied N=10, 50, 100. For case-control comparisons, the number of cases and controls are both N/2. Blue dashed lines represent type I error = 0.05.

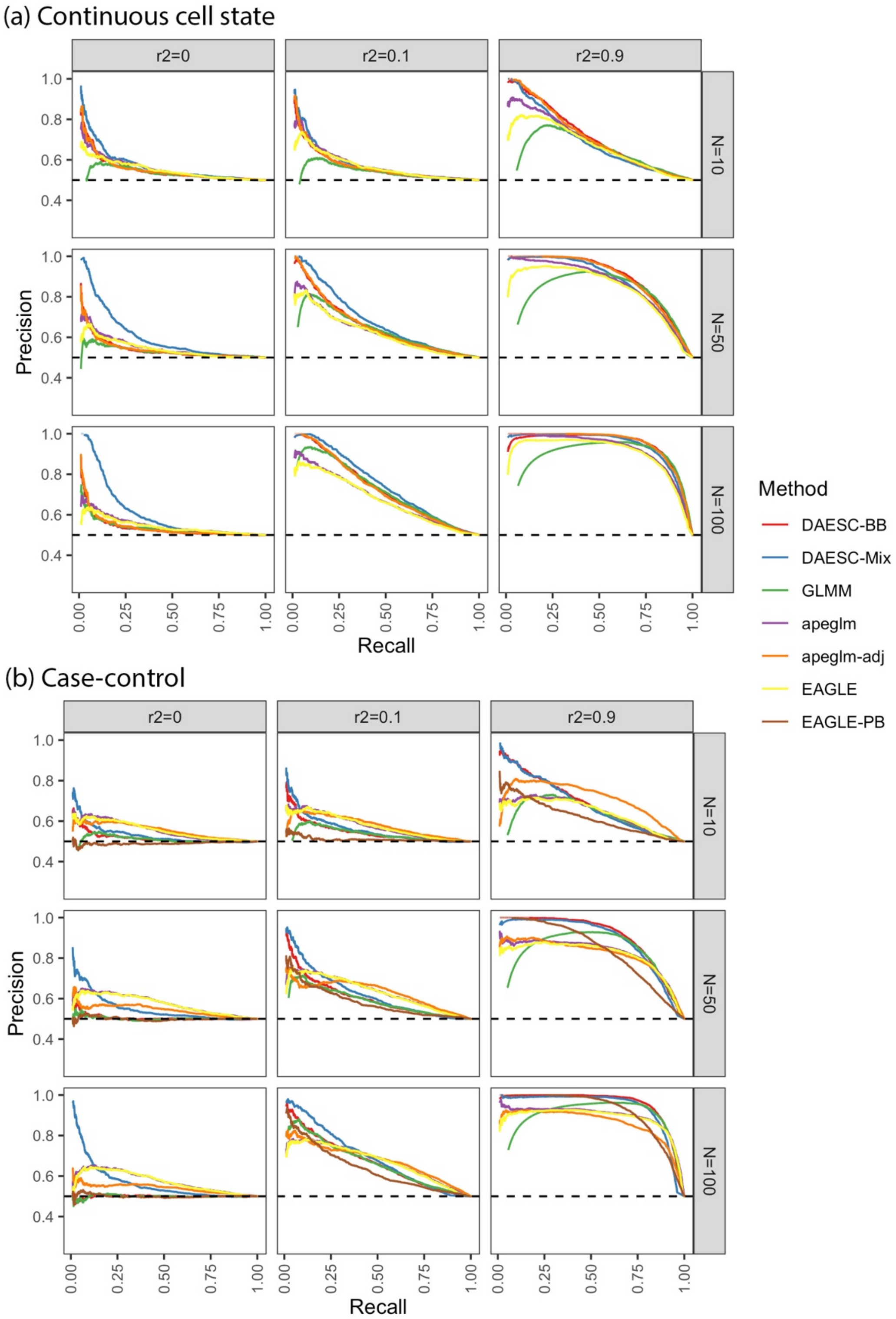

Supplementary Figure 4. Precision-recall curve for all methods compared in simulation studies. Allele-specific read counts are simulated from beta-binomial mixture model under the scenario where only one eQTL drives the ASE of a transcribed SNP (tSNP, see Methods). The linkage disequilibrium between the eQTL and the tSNP is varied to $r^2 = 0, 0.1, 0.9$, and the sample size is varied N=10, 50, 100. For case-control comparisons, the number of cases and controls are both N/2. Dashed lines represent precision = 0.5.

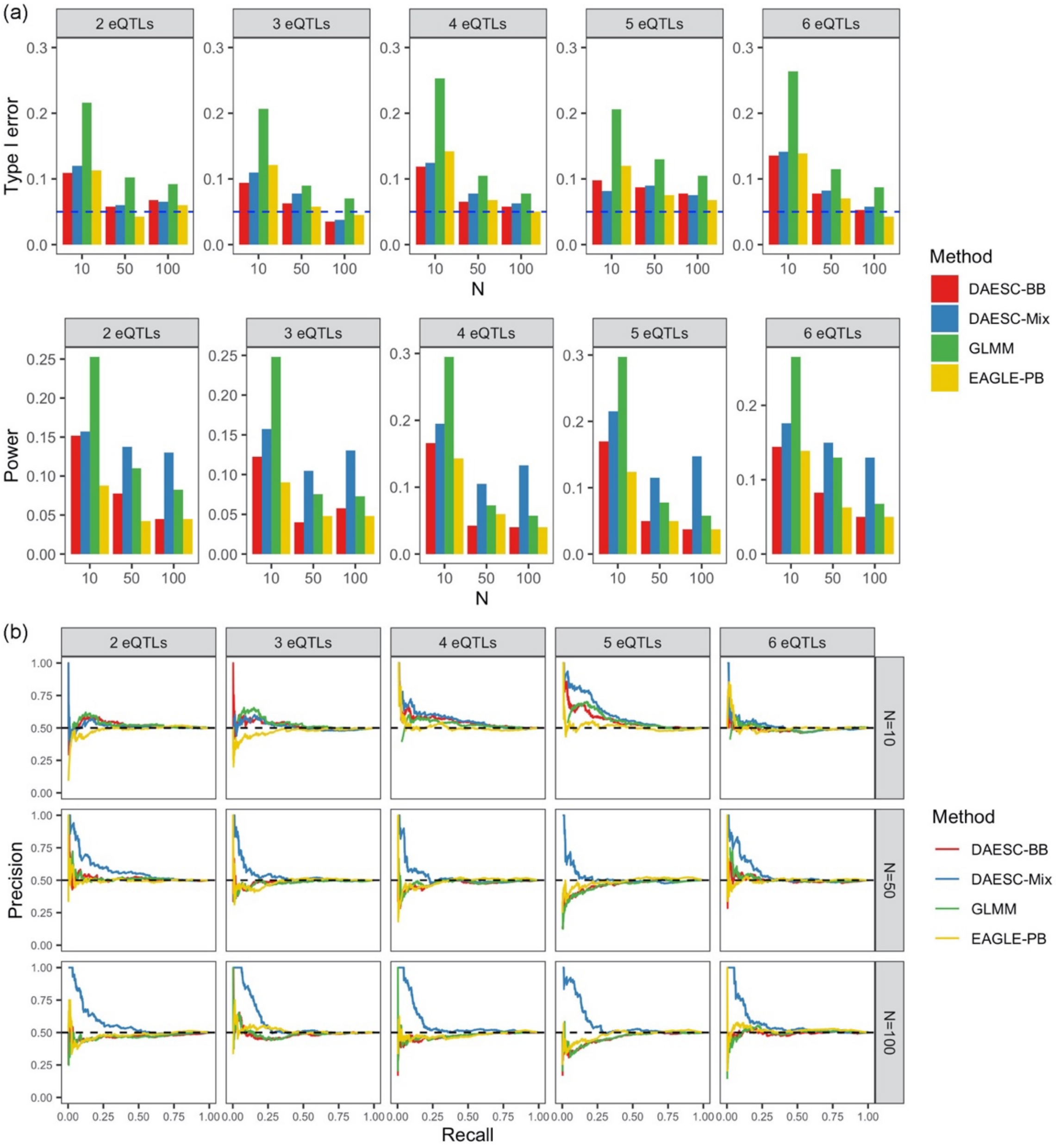

Supplementary Figure 5. Performance of four methods for differential ASE detection between disease cases and controls observed in simulation studies where multiple eQTLs drive ASE. (a) Type I error and power under significance threshold $p<0.05$ (dashed lines represent type I error = 0.05) and (b) precision-recall curves (dashed lines represent precision = 0.5). Allele-specific read counts are simulated from beta-binomial mixture model assuming multiple eQTLs drive the ASE of a tSNP. The sample size is varied to N=10, 50, 100. The number of cases and controls are both N/2.

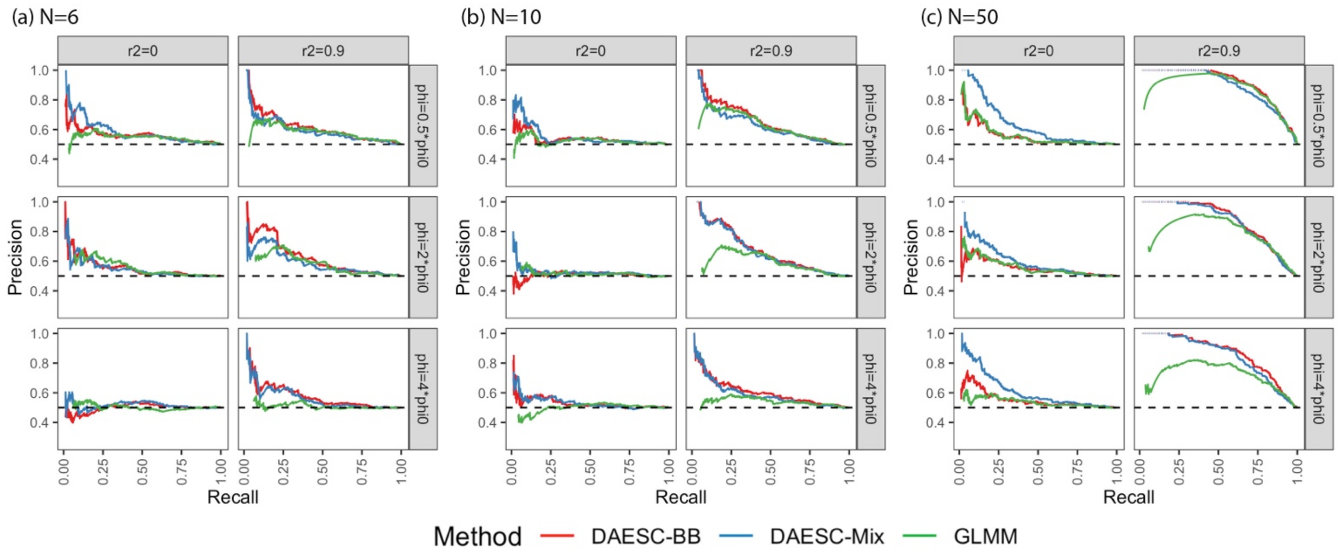

Supplementary Figure 6. Simulations under different levels of overdispersion parameter. Allele-specific read counts are simulated from beta-binomial mixture model under the scenario where only one eQTL drives the ASE of a transcribed SNP (tSNP, see Methods). Differential ASE analysis is conducted along a continuous cell state. Overdispersion parameters (ϕ or phi) are obtained by scaling the original overdispersion parameter in the candidate coefficients (ϕ_0 or phi, Supplementary Table 4). Scaling parameters (0.5, 2, or 4) reflect different levels of overdispersion. The linkage disequilibrium between the eQTL and the tSNP is varied to $r^2 = 0, 0.9$, and the sample size is varied N=6, 10, 50. Black dashed lines represent precision = 0.5.

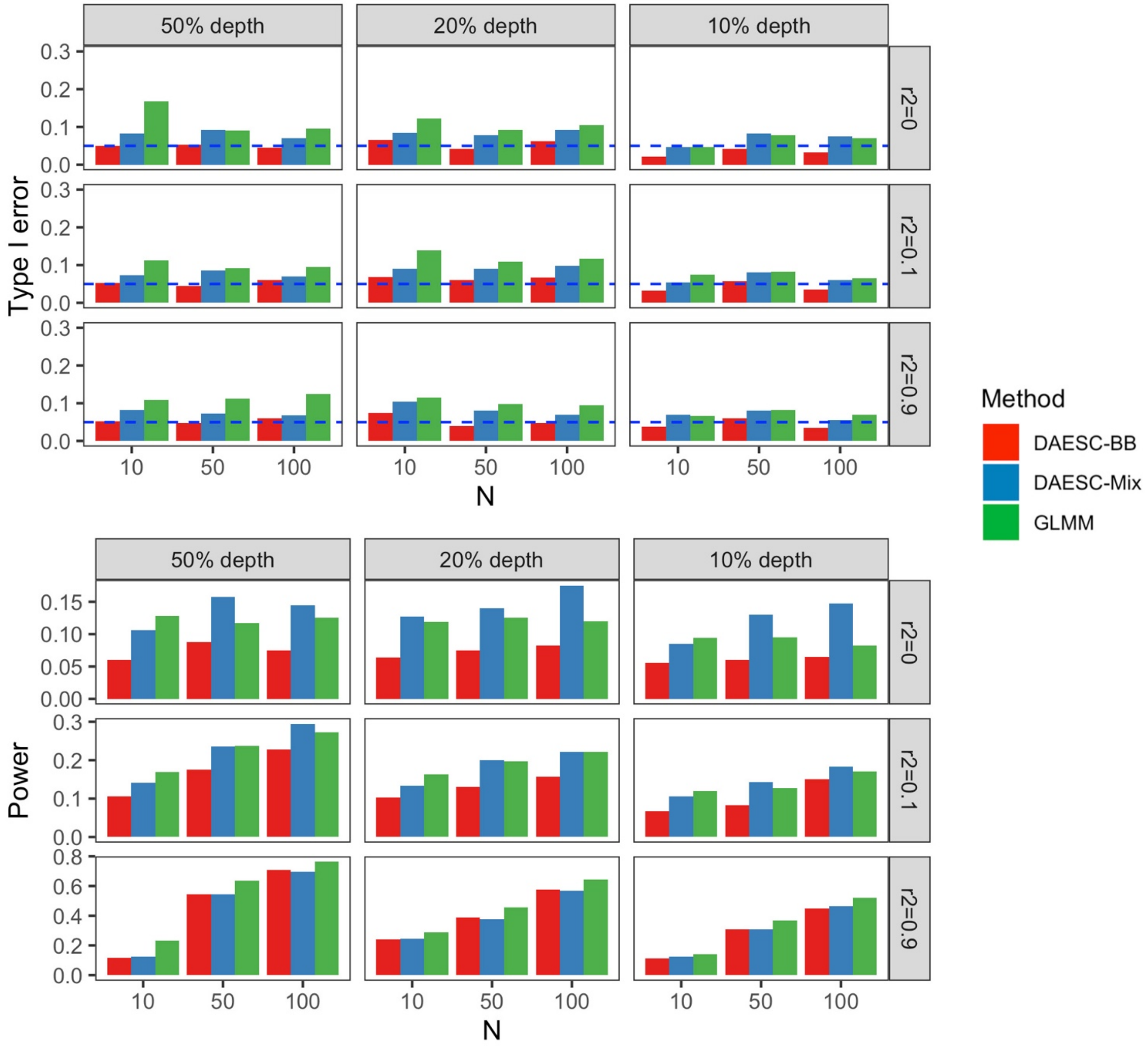

Supplementary Figure 7. Simulation studies with varying single-cell read depth. We vary the read depth to 50%, 20%, and 10% of that of the endoderm differentiation data. The original depth is 576k total reads and 27k allele-specific reads per cell. Allele-specific read counts are simulated from beta-binomial mixture model under the scenario where only one eQTL drives the ASE of a transcribed SNP (tSNP, see Methods). Differential ASE analysis is conducted along a continuous cell state. The linkage disequilibrium between the eQTL and the tSNP is varied to $r^2 = 0, 0.1, 0.9$, and the sample size is varied N=10, 50, 100. Blue dashed lines represent type I error = 0.05.

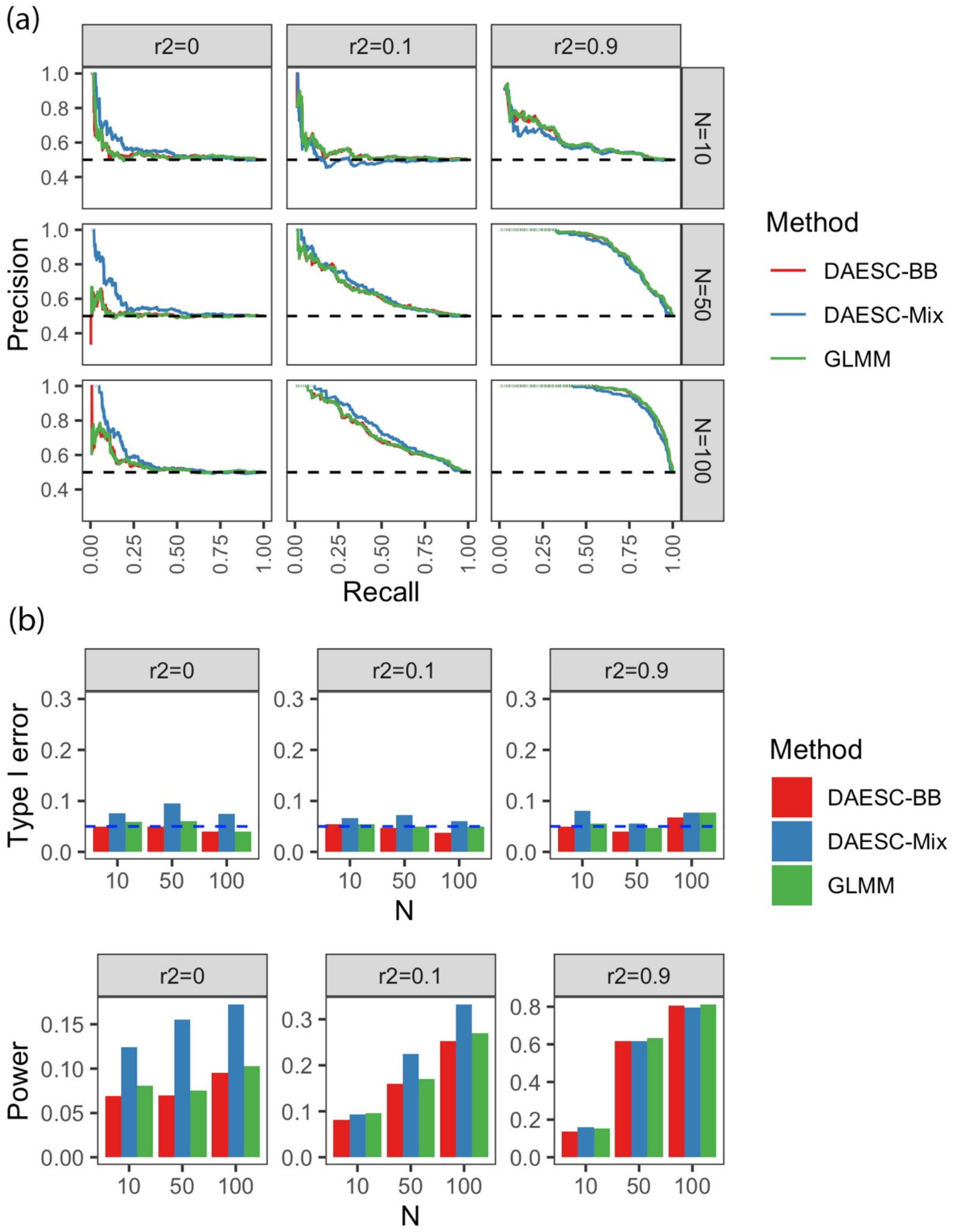

Supplementary Figure 8. Simulation studies where ASE counts are generated from binomial GLMM. (a) Precision-recall curve. Black dashed lines represent precision = 0.5. (b) Type I error and power. Blue dashed lines represent type I error = 0.05. Allele-specific read counts are simulated from mixture of binomial GLMM with donor- and cell-specific random effects. Only one eQTL is assumed to drive the ASE of a transcribed SNP (tSNP, see Methods). Differential ASE analysis is conducted along a continuous cell state. The linkage disequilibrium between the eQTL and the tSNP is varied to $r^2 = 0, 0.1, 0.9$, and the sample size is varied N=10, 50, 100.

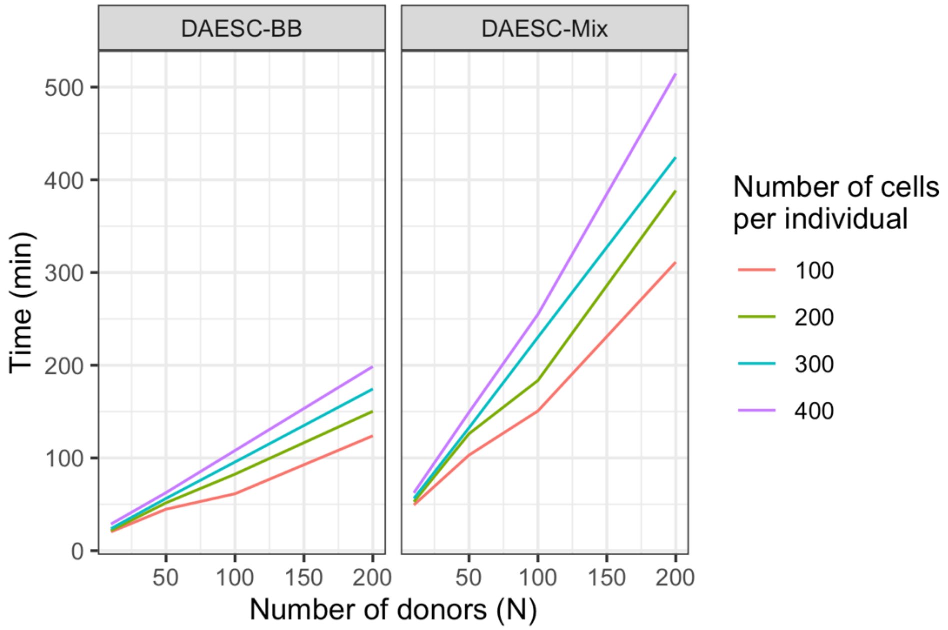

Supplementary Figure 9. Computational time for DAESC analysis of 100 genes on JHU Rockfish Computing Cluster. Allele-specific read counts are simulated from beta-binomial mixture model under the scenario where only one eQTL drives the ASE of a transcribed SNP (tSNP, see Methods). Differential ASE analysis is conducted along a continuous cell state.

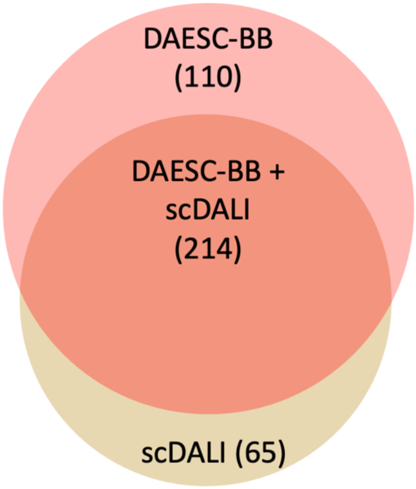

Supplementary Figure 10. Number of D-ASE genes identified by DAESC-Mix vs scDALI for the endoderm differentiation data. scDALI is implemented with 10 PCs as cell states and donor IDs as fixed-effects covariates.

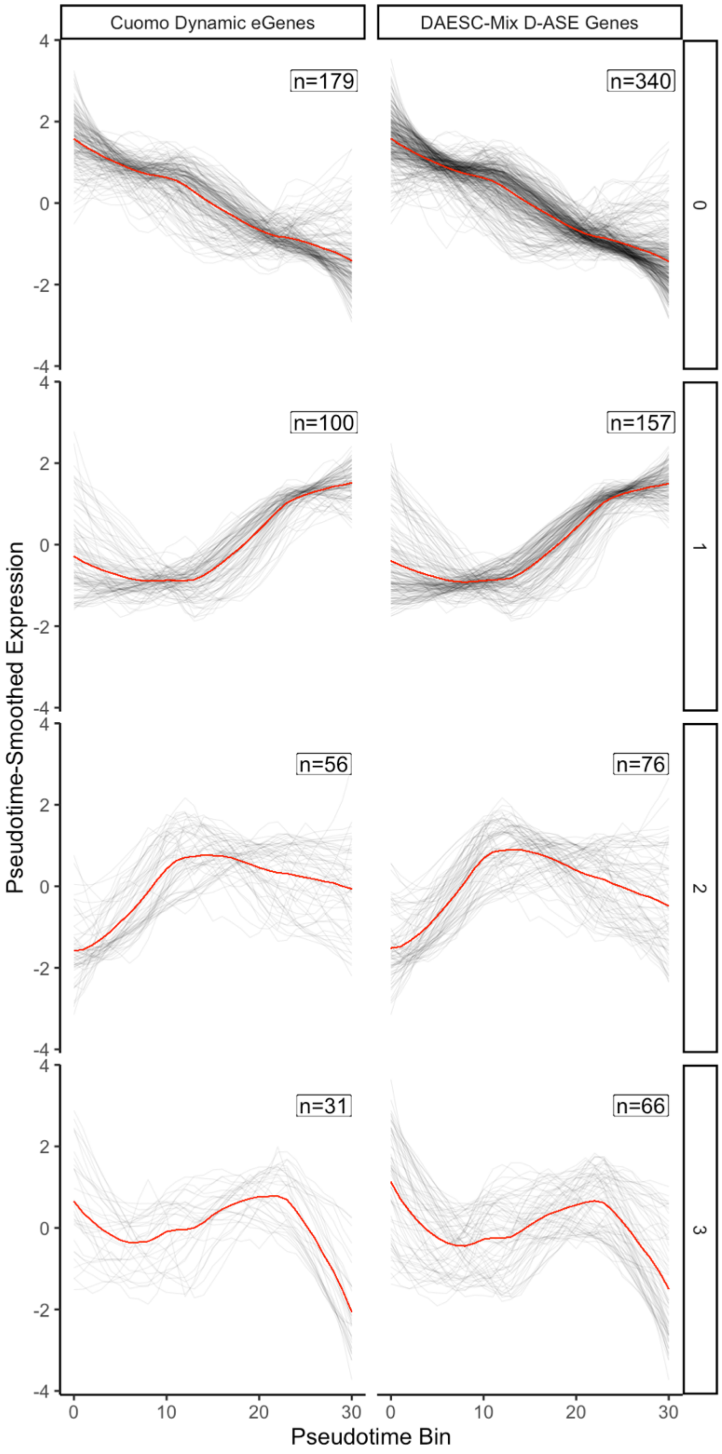

Supplementary Figure 11. Change of total expression over pseudotime for D-ASE genes identified by DAESC-Mix and dynamic eGenes reported by Cuomo et al. Each row is a cluster of patterns identified by spectral clustering. Each grey line is the trajectory of one gene and red line is the average trajectory within the cluster.

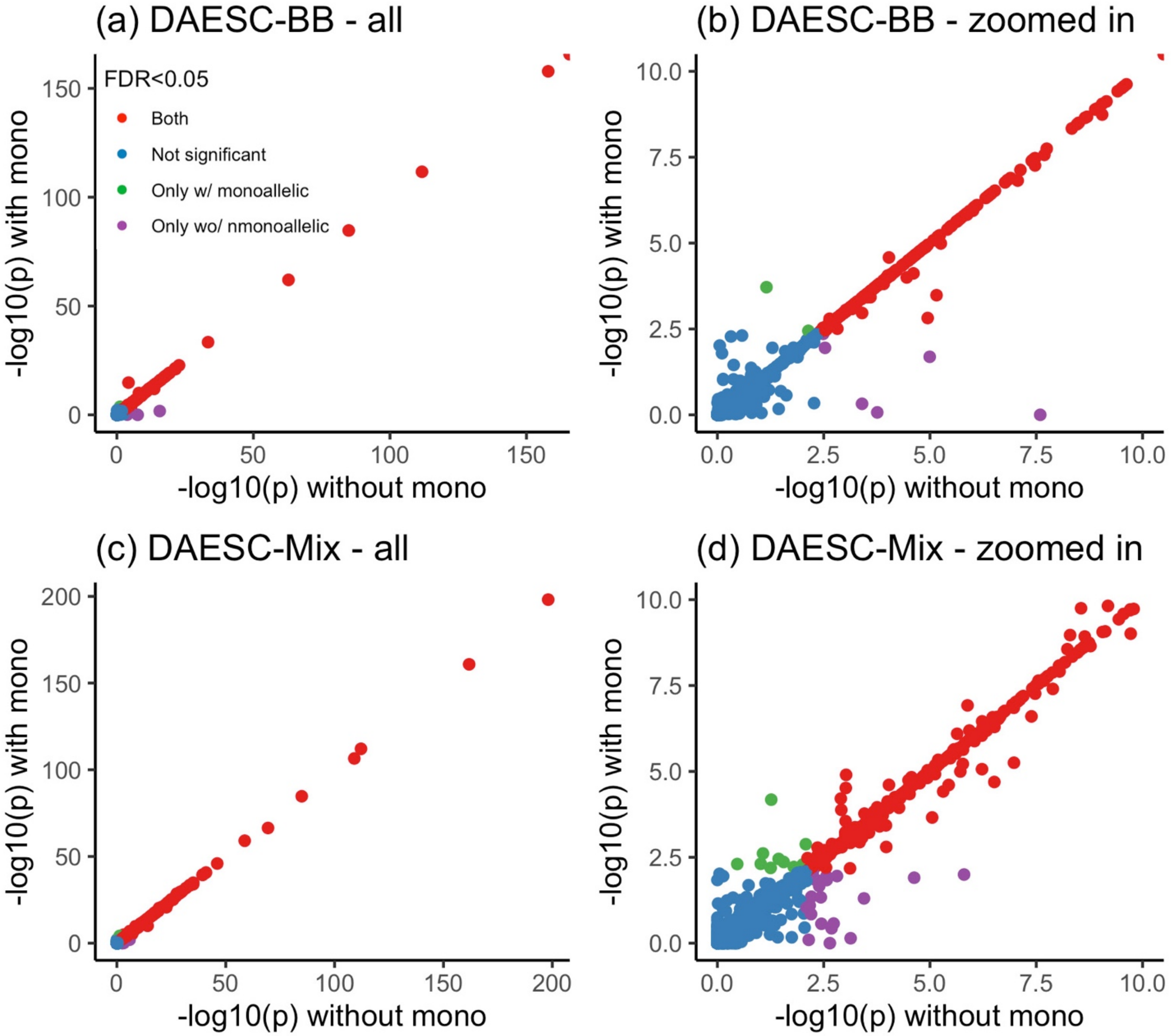

Supplementary Figure 12. DAESC p-values for the endoderm differentiation data with or without monoallelically expressed SNPs. We show all the genes in a) and c) and a zoomed-in plot (0-10) in c) and d).

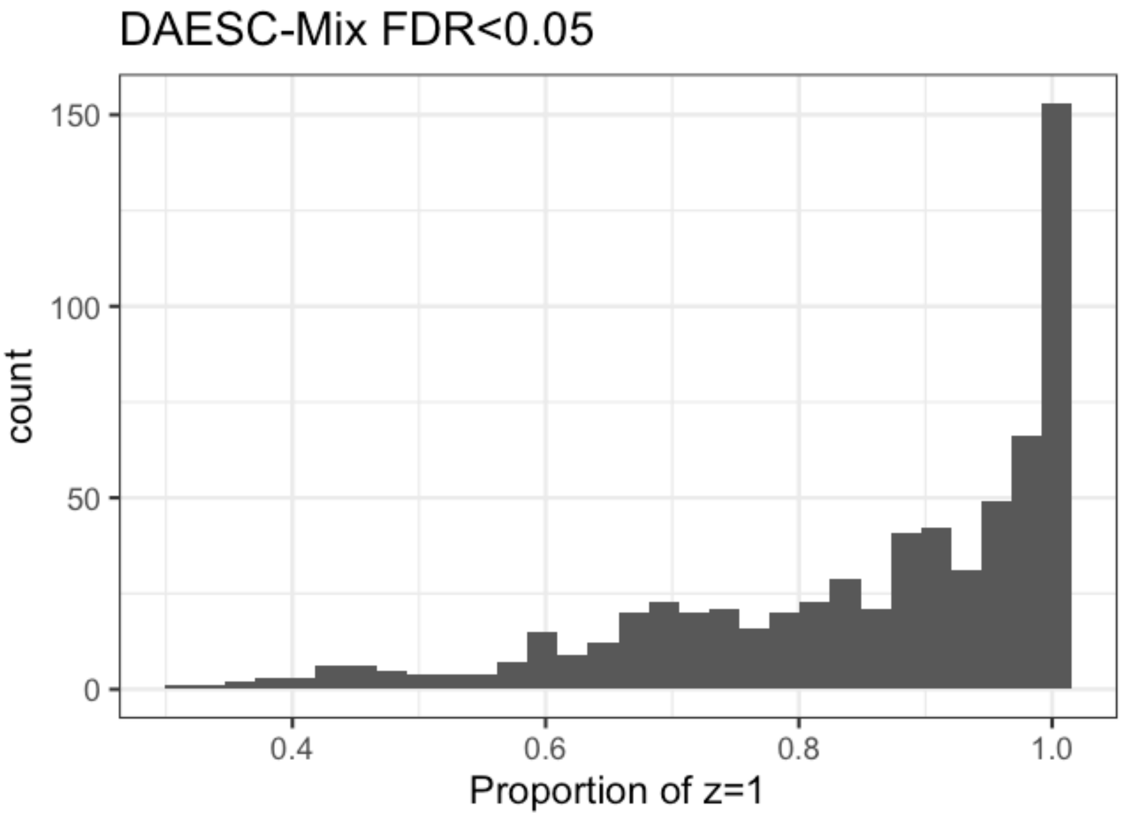

Supplementary Figure 13. Proportion of donors in the z=1 cluster learned by DAESC-Mix in the endoderm differentiation data. The histogram only plots genes with DAESC-Mix FDR<0.05. Cluster labels z=1 and z=-1 are determined posterior probability >0.5.

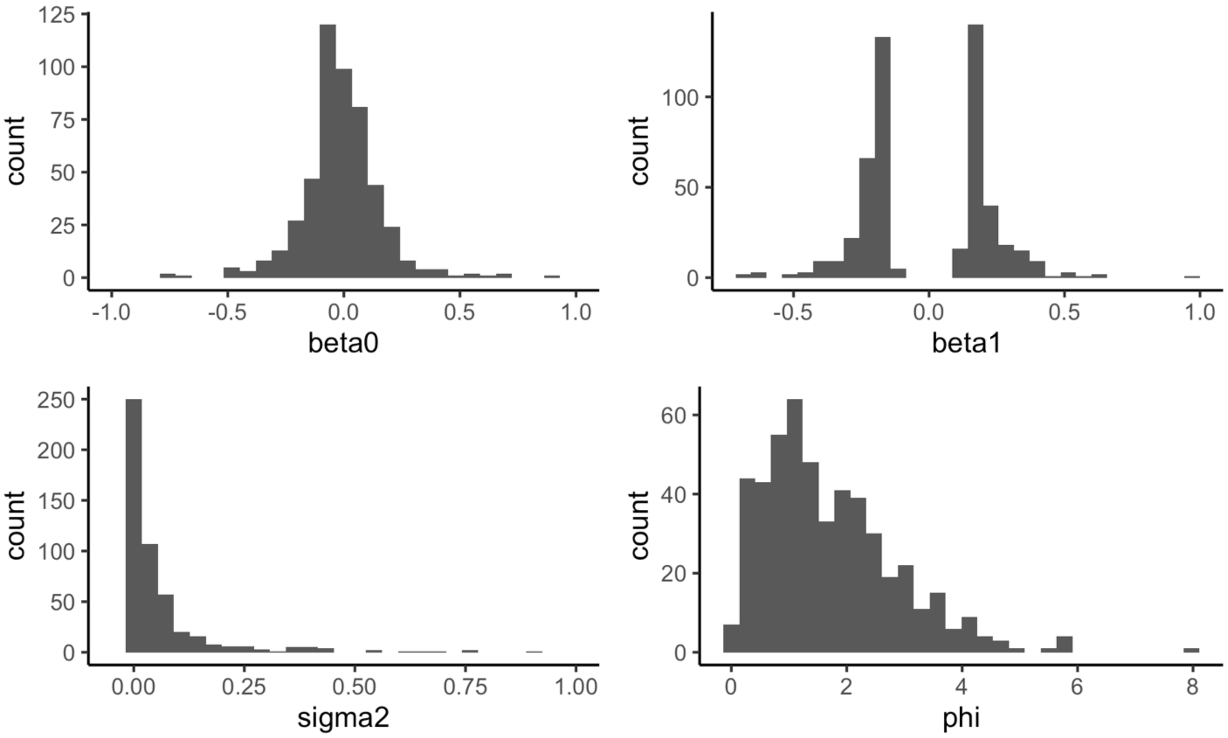

Supplementary Figure 14. Distribution of candidate simulation parameters. Exact parameters are provided in Supplementary Table 4.

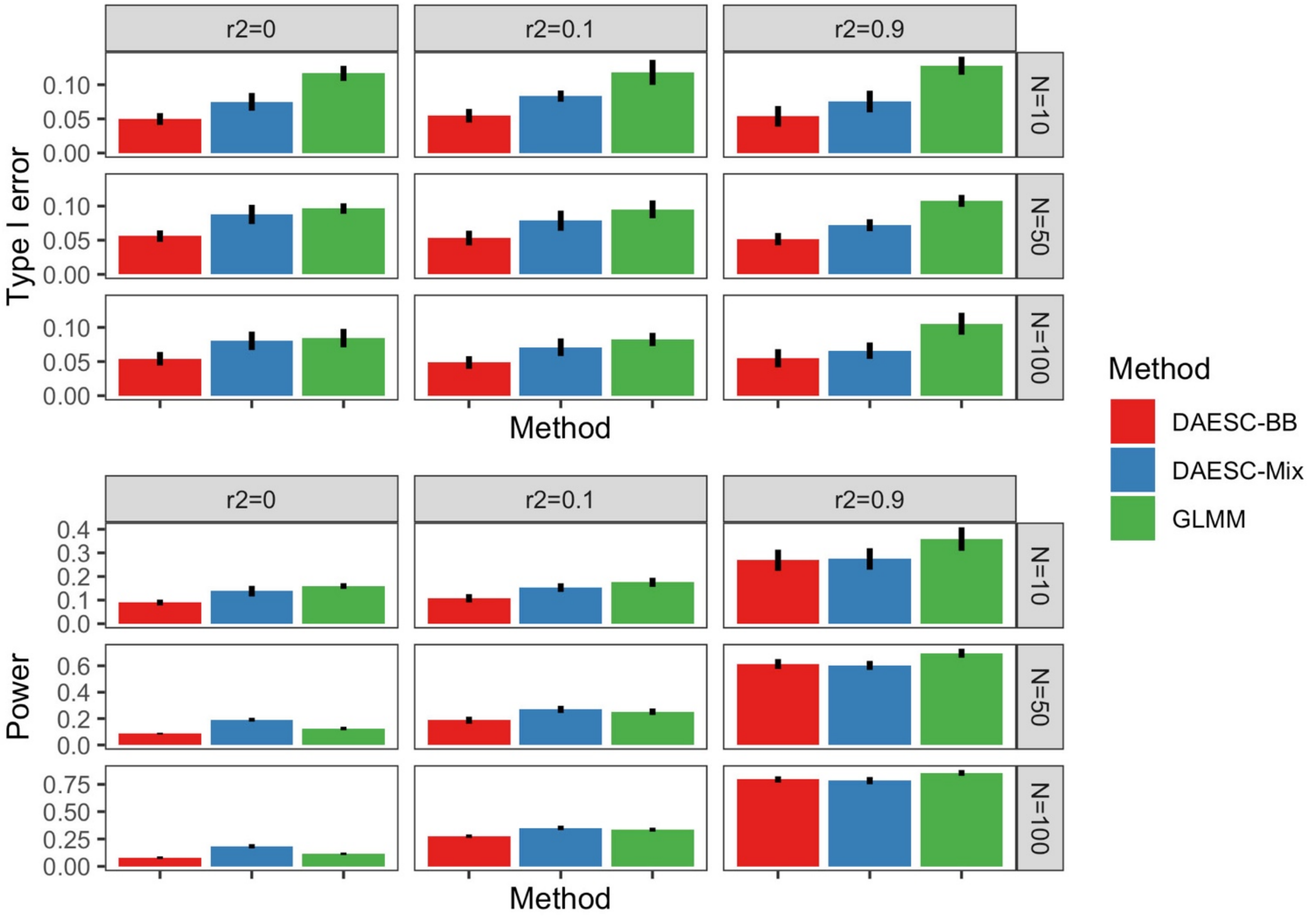

Supplementary Figure 15. The error bar of type I error and power observed in simulation studies. Allele-specific read counts are simulated from beta-binomial mixture model under the scenario where only one eQTL drives the ASE of a transcribed SNP (tSNP, see Methods). Differential ASE analysis is conducted along a continuous cell state. Error bars (black line segments) are computed across 10 replications (500 simulations each) as mean ± SD. Colored bar plots represent average across 10 replications.

Supplementary Notes

1 DAESC-BB model and inference

1.1 Model setup

For a gene or heterozygous transcribed SNP (tSNP), let y_{ij} be the alternative allele read count of individual i and cell j, and n_{ij} be the total allele-specific read count. Here $i = 1, 2, ..., N$ and $j = 1, 2, ..., J_i$. Let $\boldsymbol{x}_{ij}$ be a length-p vector of independent variables. The DAESC-BB model is formulated as follows:

$$y_{ij}|n_{ij} \sim \text{beta-binomial}(n_{ij}, \mu_{ij}, \phi) \tag{1}$$

$$\log(\mu_{ij}/(1-\mu_{ij})) = \boldsymbol{x}_{ij}^T\boldsymbol{\beta} + a_i \tag{2}$$

$$a_i \sim N(0, \sigma_a^2) \tag{3}$$

The beta-binomial distribution for y_{ij} is equivalent to $y_{ij} \sim binomial(n_{ij}, p_{ij})$, $p_{ij} \sim beta(\mu_{ij}/\phi, (1-\mu_{ij})/\phi)$. The fixed effects $\boldsymbol{\beta}$ represents ASE and dynamic ASE effects. The individual-specific random effects a_i's capture the sample repeat structure due to having multiple cells per individual.

1.2 Parameter estimation by variational EM algorithm

We use the variational EM algorithm (Wang and Blei, 2013; Blei et al, 2017) to estimate unknown parameters in model (1)-(2), treating a_i as missing data. Define $\boldsymbol{y}_i = (y_{i1}, ..., y_{iJ_i})^T$ and $\sigma(x) = 1/(1+\exp(-x))$ as the sigmoid function.

The complete data likelihood is

$$\begin{aligned}
&P(\boldsymbol{y}_1, a_1, ..., \boldsymbol{y}_N, a_N \mid \boldsymbol{\beta}, \sigma_a^2, \phi)\\
=&\prod_i \Big[\prod_j \binom{n_{ij}}{y_{ij}} \frac{B\big(\sigma(\boldsymbol{x}_{ij}^T\boldsymbol{\beta}+a_i)/\phi + y_{ij}, \sigma(\boldsymbol{x}_{ij}^T\boldsymbol{\beta}+a_i)/\phi + n_{ij} - y_{ij}\big)}{B\big(\sigma(\boldsymbol{x}_{ij}^T\boldsymbol{\beta}+a_i)/\phi, \sigma(\boldsymbol{x}_{ij}^T\boldsymbol{\beta}+a_i)/\phi\big)}\Big] (2\pi\sigma_a^2)^{-\frac{1}{2}} \exp(-\frac{a_i^2}{2\sigma_a^2})\\
\propto&(\sigma_a^2)^{-\frac{N}{2}} \prod_i \Big[\prod_j \frac{B\big(\sigma(\boldsymbol{x}_{ij}^T\boldsymbol{\beta}+a_i)/\phi + y_{ij}, \sigma(\boldsymbol{x}_{ij}^T\boldsymbol{\beta}+a_i)/\phi + n_{ij} - y_{ij}\big)}{B\big(\sigma(\boldsymbol{x}_{ij}^T\boldsymbol{\beta}+a_i)/\phi, \sigma(\boldsymbol{x}_{ij}^T\boldsymbol{\beta}+a_i)/\phi\big)}\Big] \exp(-\frac{a_i^2}{2\sigma_a^2})
\end{aligned}$$

Hence the complete-data log-likelihood is

$$\begin{aligned}
&\log P(\boldsymbol{y}_1, a_1, ..., \boldsymbol{y}_N, a_N \mid \boldsymbol{\beta}, \sigma_a^2, \phi)\\
=&\text{const} - \frac{N}{2}\log(\sigma_a^2) - \frac{1}{2\sigma_a^2}\sum_i a_i^2\\
&+\sum_i\sum_j \log B\big(\sigma(\boldsymbol{x}_{ij}^T\boldsymbol{\beta}+a_i)/\phi + y_{ij}, \sigma(\boldsymbol{x}_{ij}^T\boldsymbol{\beta}+a_i)/\phi + n_{ij} - y_{ij}\big) - \log B\big(\sigma(\boldsymbol{x}_{ij}^T\boldsymbol{\beta}+a_i)/\phi, \sigma(\boldsymbol{x}_{ij}^T\boldsymbol{\beta}+a_i)/\phi\big)
\end{aligned}$$

1.2.1 E-step

At iteration t, we approximate the conditional distribution $P(a_i \mid \boldsymbol{y}_i; \boldsymbol{\beta}_{(t)}, \sigma^2_{a,(t)}, \phi_{(t)})$ by $N(\hat{a}_{i,(t)}, \hat{\sigma}^2_{a_i,(t)})$. See section 3 for derivation of the variational approximation.

Here $\boldsymbol{\beta}_{(t)}$, $\sigma^2_{a,(t)}$ and $\phi_{(t)}$ are the current values at iteration t. The EM Q-function is expressed as

$$Q(\boldsymbol{\beta}, \sigma^2_a, \phi \mid \boldsymbol{\beta}_{(t)}, \sigma^2_{a,(t)}, \phi_{(t)}) = E_{N(\hat{a}_{i,(t)}, \hat{\sigma}^2_{a_i,(t)})}[\log P(\boldsymbol{y}_1, a_1, ..., \boldsymbol{y}_N, a_N \mid \boldsymbol{\beta}, \sigma^2_a, \phi) \mid \boldsymbol{y}_1, ..., \boldsymbol{y}_N, \boldsymbol{\beta}, \sigma^2_a, \phi].$$

The expectation does not have a closed-form solution. We use Gauss-Hermite quadrature to approximate it. Hence the Q-function can be approximated by

$$\begin{aligned}
&Q(\boldsymbol{\beta}, \sigma^2_a, \phi \mid \boldsymbol{\beta}_{(t)}, \sigma^2_{a,(t)}, \phi_{(t)}) \\
=&\text{const} - \frac{N}{2}\log(\sigma^2_a) - \frac{1}{2\sigma^2_a}\sum_i \hat{a}^2_{i,(t)} + \hat{\sigma}^2_{a_i,(t)} \\
&+ \sum_m w_m \{ \sum_i \sum_j \log B\big(\sigma(\boldsymbol{x}_{ij}^T\boldsymbol{\beta} + \hat{a}_{i,(t)} + \hat{\sigma}_{a_i,(t)} z_m)/\phi + y_{ij}, \sigma(\boldsymbol{x}_{ij}^T\boldsymbol{\beta} + \hat{a}_{i,(t)} + \hat{\sigma}_{a_i,(t)} z_m)/\phi + n_{ij} - y_{ij}\big) \\
&\quad - \log B\big(\sigma(\boldsymbol{x}_{ij}^T\boldsymbol{\beta} + \hat{a}_{i,(t)} + \hat{\sigma}_{a_i,(t)} z_m)/\phi, \sigma(\boldsymbol{x}_{ij}^T\boldsymbol{\beta} + \hat{a}_{i,(t)} + \hat{\sigma}_{a_i,(t)} z_m)/\phi\big) \}
\end{aligned}$$

Here (z_m, w_m), $m = 1, ..., M$ are the nodes and weights of a Gauss-Hermite quadrature for standard normal distribution. In practice, we found $M = 3$ nodes is sufficient for approximating the Q function.

1.2.2 M-step

The update for σ^2_a has a closed form:

$$\sigma^2_{a,(t+1)} = \frac{1}{N}\sum_i \hat{a}^2_{i,(t)} + \hat{\sigma}^2_{a_i,(t)}$$

The update for $\boldsymbol{\beta}$ and ϕ is obtained by maximizing the Q function using Newton-Raphson.

2 DAESC-Mix model and inference

2.1 Model setup

DAESC-Mix is an extension of DAESC-BB incorporating a latent variable δ_i for implicit haplotype phasing. The model is formulated as follows:

$$y_{ij} | n_{ij} \sim \text{beta-binomial}(n_{ij}, \mu_{ij}, \phi) \tag{4}$$

$$\log(\mu_{ij}/(1-\mu_{ij})) = (2\delta_i - 1)\boldsymbol{x}_{ij}^T\boldsymbol{\beta} + a_i \tag{5}$$

$$a_i \sim N(0, \sigma^2_a), \quad \delta_i \sim \text{Bernoulli}(\pi_0) \tag{6}$$

The variable δ_i models the scenario where ASE is caused by one regulatory SNP (rSNP). When $\delta_i = 1$, the alternative allele of the eQTL and the alternative allele of the tSNP are on the same haplotype, and the reference alleles of the two SNPs are on the same haplotype. When $\delta_i = 0$, the alternative allele of the eQTL and the reference allele of the tSNP are on the same haplotype, and vice versa (Figure 1).

2.2 Parameter estimation by variational EM algorithm

We treat a_i and δ_i as missing data. The complete-data likelihood is

$$
\begin{aligned}
&P(\boldsymbol{y}_1, a_1, \delta_1, ..., \boldsymbol{y}_N, a_N, \delta_N \mid \boldsymbol{\beta}, \sigma_a^2, \phi, \pi_0) \\
=&\prod_i \Big[\pi_0 \prod_j \binom{n_{ij}}{y_{ij}} \frac{B\big(\sigma(\boldsymbol{x}_{ij}^T\boldsymbol{\beta}+a_i)/\phi + y_{ij}, \sigma(\boldsymbol{x}_{ij}^T\boldsymbol{\beta}+a_i)/\phi + n_{ij} - y_{ij}\big)}{B\big(\sigma(\boldsymbol{x}_{ij}^T\boldsymbol{\beta}+a_i)/\phi, \sigma(\boldsymbol{x}_{ij}^T\boldsymbol{\beta}+a_i)/\phi\big)}\Big]^{\delta_i} \times \\
&\Big[(1-\pi_0) \prod_j \binom{n_{ij}}{y_{ij}} \frac{B\big(\sigma(-\boldsymbol{x}_{ij}^T\boldsymbol{\beta}+a_i)/\phi + y_{ij}, \sigma(-\boldsymbol{x}_{ij}^T\boldsymbol{\beta}+a_i)/\phi + n_{ij} - y_{ij}\big)}{B\big(\sigma(-\boldsymbol{x}_{ij}^T\boldsymbol{\beta}+a_i)/\phi, \sigma(-\boldsymbol{x}_{ij}^T\boldsymbol{\beta}+a_i)/\phi\big)}\Big]^{1-\delta_i} (2\pi\sigma_a^2)^{-\frac{1}{2}} \exp(-\frac{a_i^2}{2\sigma_a^2}) \\
\propto&(\sigma_a^2)^{-\frac{N}{2}} \prod_i \Big[\pi_0 \prod_j \frac{B\big(\sigma(\boldsymbol{x}_{ij}^T\boldsymbol{\beta}+a_i)/\phi + y_{ij}, \sigma(\boldsymbol{x}_{ij}^T\boldsymbol{\beta}+a_i)/\phi + n_{ij} - y_{ij}\big)}{B\big(\sigma(\boldsymbol{x}_{ij}^T\boldsymbol{\beta}+a_i)/\phi, \sigma(\boldsymbol{x}_{ij}^T\boldsymbol{\beta}+a_i)/\phi\big)}\Big]^{\delta_i} \times \\
&\Big[(1-\pi_0) \prod_j \frac{B\big(\sigma(-\boldsymbol{x}_{ij}^T\boldsymbol{\beta}+a_i)/\phi + y_{ij}, \sigma(-\boldsymbol{x}_{ij}^T\boldsymbol{\beta}+a_i)/\phi + n_{ij} - y_{ij}\big)}{B\big(\sigma(-\boldsymbol{x}_{ij}^T\boldsymbol{\beta}+a_i)/\phi, \sigma(-\boldsymbol{x}_{ij}^T\boldsymbol{\beta}+a_i)/\phi\big)}\Big]^{1-\delta_i} \exp(-\frac{a_i^2}{2\sigma_a^2})
\end{aligned}
$$

The complete-data log-likelihood is

$$
\begin{aligned}
&\log P(\boldsymbol{y}_1, a_1, \delta_1, ..., \boldsymbol{y}_N, a_N, \delta_N \mid \boldsymbol{\beta}, \sigma_a^2, \phi, \pi_0) \\
=&\text{const} - \frac{N}{2}\log(\sigma_a^2) + \sum_i \Big\{ -\frac{a_i^2}{2\sigma_a^2} \\
+&\delta_i \Big[\log \pi_0 + \sum_j \log B\big(\sigma(\boldsymbol{x}_{ij}^T\boldsymbol{\beta}+a_i)/\phi + y_{ij}, \sigma(\boldsymbol{x}_{ij}^T\boldsymbol{\beta}+a_i)/\phi + n_{ij} - y_{ij}\big) \\
-&\sum_j \log B\big(\sigma(\boldsymbol{x}_{ij}^T\boldsymbol{\beta}+a_i)/\phi, \sigma(\boldsymbol{x}_{ij}^T\boldsymbol{\beta}+a_i)/\phi\big)\Big] \\
+&(1-\delta_i)\Big[\log(1-\pi_0) + \sum_j \log B\big(\sigma(-\boldsymbol{x}_{ij}^T\boldsymbol{\beta}+a_i)/\phi + y_{ij}, \sigma(-\boldsymbol{x}_{ij}^T\boldsymbol{\beta}+a_i)/\phi + n_{ij} - y_{ij}\big) \\
-&\sum_j \log B\big(\sigma(-\boldsymbol{x}_{ij}^T\boldsymbol{\beta}+a_i)/\phi, \sigma(-\boldsymbol{x}_{ij}^T\boldsymbol{\beta}+a_i)/\phi\big)\Big]\Big\}
\end{aligned}
$$

2.2.1 E-step

At iteration t, we approximate the posterior distribution $P(a_i, \delta_i \mid \boldsymbol{y}_i; \boldsymbol{\beta}_{(t)}, \sigma^2_{a,(t)}, \phi_{(t)}, \pi_{0,(t)})$ using variational inference (Blei et al, 2017). Using the mean-field approximation $q(a_i, \delta_i) = q(a_i)q(\delta_i)$, we update $q(\delta_i)$ and $q(a_i)$ iteratively as follows. The update for $q(\delta_i)$ is

$$
\begin{aligned}
&\log q(\delta_i) = E_{q(a_i)}[\log P(a_i, \delta_i \mid \boldsymbol{y}_i, \boldsymbol{\beta}_{(t)}, \sigma^2_{a,(t)}, \phi_{(t)})] \\
=&\text{const} + \delta_i \Big[\log \pi_0 + \sum_j \int \log B\big(\sigma(\boldsymbol{x}_{ij}^T\boldsymbol{\beta}_{(t)}+a_i)/\phi_{(t)} + y_{ij}, \sigma(\boldsymbol{x}_{ij}^T\boldsymbol{\beta}_{(t)}+a_i)/\phi_{(t)} + n_{ij} - y_{ij}\big) q(a_i) da_i \\
-&\sum_j \int \log B\big(\sigma(\boldsymbol{x}_{ij}^T\boldsymbol{\beta}_{(t)}+a_i)/\phi_{(t)}, \sigma(\boldsymbol{x}_{ij}^T\boldsymbol{\beta}_{(t)}+a_i)/\phi_{(t)}\big) q(a_i) da_i\Big] \\
+&(1-\delta_i)\Big[\log(1-\pi_0) + \sum_j \int \log B\big(\sigma(-\boldsymbol{x}_{ij}^T\boldsymbol{\beta}_{(t)}+a_i)/\phi_{(t)} + y_{ij}, \sigma(-\boldsymbol{x}_{ij}^T\boldsymbol{\beta}_{(t)}+a_i)/\phi_{(t)} + n_{ij} - y_{ij}\big) q(a_i) da_i \\
-&\sum_j \int \log B\big(\sigma(-\boldsymbol{x}_{ij}^T\boldsymbol{\beta}_{(t)}+a_i)/\phi_{(t)}, \sigma(-\boldsymbol{x}_{ij}^T\boldsymbol{\beta}_{(t)}+a_i)/\phi_{(t)}\big) q(a_i) da_i\Big]
\end{aligned}
$$

The integrals are approximated by Gauss-Hermite quadrature (see Section 1.2.1 for details). The resulting distribution is a bernoulli distribution, denoted by $\text{Ber}(\pi_{i,(t)})$. The variational update for a_i is

$$\log q(a_i) = E_{q(\delta_i)}[\log P(a_i, \delta_i \mid \boldsymbol{y}_i, \boldsymbol{\beta}_{(t)}, \sigma^2_{a,(t)}, \phi_{(t)})]$$
$$= \text{const} - \frac{a_i^2}{2\sigma^2_{a,(t)}} + \pi_{i,(t)} \Big[\sum_j \log B\big(\sigma(\boldsymbol{x}_{ij}^T \boldsymbol{\beta}_{(t)} + a_i)/\phi_{(t)} + y_{ij}, \sigma(\boldsymbol{x}_{ij}^T \boldsymbol{\beta}_{(t)} + a_i)/\phi_{(t)} + n_{ij} - y_{ij}\big)$$
$$- \sum_j \log B\big(\sigma(\boldsymbol{x}_{ij}^T \boldsymbol{\beta}_{(t)} + a_i)/\phi_{(t)}, \sigma(\boldsymbol{x}_{ij}^T \boldsymbol{\beta}_{(t)} + a_i)/\phi_{(t)}\big)\Big]$$
$$+(1 - \pi_{i,(t)}) \Big[\sum_j \log B\big(\sigma(-\boldsymbol{x}_{ij}^T \boldsymbol{\beta}_{(t)} + a_i)/\phi_{(t)} + y_{ij}, \sigma(-\boldsymbol{x}_{ij}^T \boldsymbol{\beta}_{(t)} + a_i)/\phi_{(t)} + n_{ij} - y_{ij}\big)$$
$$- \sum_j \log B\big(\sigma(-\boldsymbol{x}_{ij}^T \boldsymbol{\beta}_{(t)} + a_i)/\phi_{(t)}, \sigma(-\boldsymbol{x}_{ij}^T \boldsymbol{\beta}_{(t)} + a_i)/\phi_{(t)}\big)\Big]$$

This update has no closed form, but we approximate by a normal distribution $N(\hat{a}_{i,(t)}, \hat{\sigma}^2_{a_i,(t)})$, as in Laplace variational inference (Wang and Blei, 2013). See section 3.2 for details.

2.2.2 M-step

- Update π_0 by $\pi_{0,(t+1)} = \frac{1}{N} \sum\limits_i \pi_{i,(t)}$.
- Update σ_a by $\sigma^2_{a,(t+1)} = \frac{1}{N} \sum_i \hat{a}^2_{i,(t)} + \hat{\sigma}^2_{a_i,(t)}$.
- Similar to section 1.2.2, update $\boldsymbol{\beta}$ and ϕ by numerical maximization of $E_{q(\delta_i,a_i)}[\log P(\boldsymbol{y}_1, a_1, \delta_1, ..., \boldsymbol{y}_N, a_N, \delta_N \mid \boldsymbol{\beta}, \sigma^2_a, \phi, \pi_0)]$, which is the complete data log likelihood integrated over variation distribution $q(\delta_i, a_i)$. The integration over $q(a_i)$ is conducted numerically using Gaussian-Hermite quadrature.

3 Approximating posterior distribution $P(a_i \mid \boldsymbol{y}_i)$ in the E-step

3.1 DAESC-BB

In the DAESC-BB model, the joint distribution of $\boldsymbol{y}_i$ and a_i is

$$P(\boldsymbol{y}_i, a_i \mid \boldsymbol{\beta}, \sigma^2_a, \phi) \propto \prod_j \Big[\frac{B\big(\sigma(\boldsymbol{x}_{ij}^T\boldsymbol{\beta} + a_i)/\phi + y_{ij}, \sigma(\boldsymbol{x}_{ij}^T\boldsymbol{\beta} + a_i)/\phi + n_{ij} - y_{ij}\big)}{B\big(\sigma(\boldsymbol{x}_{ij}^T\boldsymbol{\beta} + a_i)/\phi, \sigma(\boldsymbol{x}_{ij}^T\boldsymbol{\beta} + a_i)/\phi\big)}\Big] \exp(-\frac{a_i^2}{2\sigma_a^2})$$

Define $\boldsymbol{X}_i = (\boldsymbol{x}_{i1}, ..., \boldsymbol{x}_{iJ_i})^T$, $\boldsymbol{y}_i = (y_{i1}, ..., y_{iJ_i})^T$ and $\boldsymbol{n}_i = (n_{i1}, ..., n_{iJ_i})^T$. Define $f_{\boldsymbol{\beta},\sigma^2_a,\phi,\boldsymbol{X}_i,\boldsymbol{y}_i,\boldsymbol{n}_i}(a_i)$ as the log joint distribution, i.e.

$$f_{\boldsymbol{\beta},\sigma^2_a,\phi,\boldsymbol{X}_i,\boldsymbol{y}_i,\boldsymbol{n}_i}(a_i) = \sum_j \log B\big(\sigma(\boldsymbol{x}_{ij}^T\boldsymbol{\beta} + a_i)/\phi + y_{ij}, [1 - \sigma(\boldsymbol{x}_{ij}^T\boldsymbol{\beta} + a_i)]/\phi + n_{ij} - y_{ij}\big) -$$
$$\sum_j \log B\big(\sigma(\boldsymbol{x}_{ij}^T\boldsymbol{\beta} + a_i)/\phi, [1 - \sigma(\boldsymbol{x}_{ij}^T\boldsymbol{\beta} + a_i)]/\phi\big) - \frac{a_i^2}{2\sigma_a^2}$$
$$= \sum_j \log B\big(\mu_{ij}/\phi + y_{ij}, (1-\mu_{ij})/\phi + n_{ij} - y_{ij}\big) -$$
$$\sum_j \log B\big(\mu_{ij}/\phi, (1-\mu_{ij})/\phi\big) - \frac{a_i^2}{2\sigma_a^2}$$
$$= \sum_j \log\Gamma\big(\mu_{ij}/\phi + y_{ij}\big) + \sum_j \log\Gamma\big((1-\mu_{ij})/\phi + n_{ij} - y_{ij}\big) - \sum_j \log\Gamma\big(1/\phi + n_{ij}\big) -$$
$$\sum_j \log\Gamma\big(\mu_{ij}/\phi\big) - \sum_j \log\Gamma\big((1-\mu_{ij})/\phi\big) + J_i \log\Gamma\big(1/\phi\big) - \frac{a_i^2}{2\sigma_a^2}$$

Here $\mu_{ij} = \sigma(\boldsymbol{x}_{ij}^T\boldsymbol{\beta} + a_i)$. To approximate $P(a_i \mid \boldsymbol{y}_i; \boldsymbol{\beta}, \sigma_a^2, \phi)$, we derived the Taylor expansion of $f_{\boldsymbol{\beta},\sigma_a^2,\phi,\boldsymbol{X}_i,\boldsymbol{y}_i,\boldsymbol{n}_i}(a_i)$. Denote by $\hat{a}_i$ the value that maximizes $f_{\boldsymbol{\beta},\sigma_a^2,\phi,\boldsymbol{X}_i,\boldsymbol{y}_i,\boldsymbol{n}_i}(a_i)$, we have

$$f_{\boldsymbol{\beta},\sigma_a^2,\phi,\boldsymbol{X}_i,\boldsymbol{y}_i,\boldsymbol{n}_i}(a_i) \approx f_{\boldsymbol{\beta},\sigma_a^2,\phi,\boldsymbol{X}_i,\boldsymbol{y}_i,\boldsymbol{n}_i}(\hat{a}_i) + \frac{1}{2}f''_{\boldsymbol{\beta},\sigma_a^2,\phi,\boldsymbol{X}_i,\boldsymbol{y}_i,\boldsymbol{n}_i}(\hat{a}_i)(a_i - \hat{a}_i)^2$$

Hence the posterior distribution $P(a_i \mid \boldsymbol{y}_i, \boldsymbol{\beta}, \sigma_a^2, \phi)$ can be approximated by $N(\hat{a}_i, \hat{\sigma}_{a_i}^2)$, where $\hat{\sigma}_{a_i}^2 = |f''_{\boldsymbol{\beta},\sigma_a^2,\phi,\boldsymbol{X}_i,\boldsymbol{y}_i,\boldsymbol{n}_i}(\hat{a}_i)|^{-1}$.

Now we derive the derivatives of $f_{\boldsymbol{\beta},\sigma_a^2,\phi,\boldsymbol{X}_i,\boldsymbol{y}_i,\boldsymbol{n}_i}(a_i)$ and the Newton-Raphson algorithm to obtain $\hat{a}_i$.

$$\begin{aligned}
f'_{\boldsymbol{\beta},\sigma_a^2,\phi,\boldsymbol{X}_i,\boldsymbol{y}_i,\boldsymbol{n}_i}(a_i) =& \sum_j \Big[\psi\Big(\frac{\mu_{ij}}{\phi} + y_{ij}\Big) - \psi\Big(\frac{1-\mu_{ij}}{\phi} + n_{ij} - y_{ij}\Big) - \psi\Big(\frac{\mu_{ij}}{\phi}\Big) + \psi\Big(\frac{1-\mu_{ij}}{\phi}\Big)\Big]\mu_{ij}(1-\mu_{ij})/\phi - \frac{a_i}{\sigma_a^2} \\
f''_{\boldsymbol{\beta},\sigma_a^2,\phi,\boldsymbol{X}_i,\boldsymbol{y}_i,\boldsymbol{n}_i}(a_i) =& \sum_j \Big\{\Big[\psi_1\Big(\frac{\mu_{ij}}{\phi} + y_{ij}\Big) + \psi_1\Big(\frac{1-\mu_{ij}}{\phi} + n_{ij} - y_{ij}\Big) - \psi_1\Big(\frac{\mu_{ij}}{\phi}\Big) - \psi_1\Big(\frac{1-\mu_{ij}}{\phi}\Big)\Big]\mu_{ij}^2(1-\mu_{ij})^2/\phi^2 + \\
& \Big[\psi\Big(\frac{\mu_{ij}}{\phi} + y_{ij}\Big) - \psi\Big(\frac{1-\mu_{ij}}{\phi} + n_{ij} - y_{ij}\Big) - \psi\Big(\frac{\mu_{ij}}{\phi}\Big) + \psi\Big(\frac{1-\mu_{ij}}{\phi}\Big)\Big](1-2\mu_{ij})\mu_{ij}(1-\mu_{ij})/\phi\Big\} - \frac{1}{\sigma_a^2}
\end{aligned}$$

Here $\psi(x) = \frac{d}{dx}\log\Gamma(x)$ is the digamma function and $\psi_1(x) = \frac{d^2}{dx^2}\log\Gamma(x)$ is the trigamma function. To obtain $\hat{a}_i$, we use a modified Newton-Raphson method. At iteration k, we update a_i as follows

$$a_i^{k+1} = a_i^k - \tau f'(a_i^k)/f''(a_i^k)$$

By default, we set $\tau = 0.9$.

3.2 DAESC-Mix

In the E-step for DAESC-Mix, the log variational distribution for a_i is

$$\begin{aligned}
& h(a_i) = \log q(a_i) \\
=& \text{const} - \frac{a_i^2}{2\sigma_a^2} + \pi_i\Big[\sum_j \log B\big(\sigma(\boldsymbol{x}_{ij}^T\boldsymbol{\beta} + a_i)/\phi + y_{ij}, \sigma(\boldsymbol{x}_{ij}^T\boldsymbol{\beta} + a_i)/\phi + n_{ij} - y_{ij}\big) \\
-& \sum_j \log B\big(\sigma(\boldsymbol{x}_{ij}^T\boldsymbol{\beta} + a_i)/\phi, \sigma(\boldsymbol{x}_{ij}^T\boldsymbol{\beta} + a_i)/\phi\big)\Big] \\
+& (1-\pi_i)\Big[\sum_j \log B\big(\sigma(-\boldsymbol{x}_{ij}^T\boldsymbol{\beta} + a_i)/\phi + y_{ij}, \sigma(-\boldsymbol{x}_{ij}^T\boldsymbol{\beta} + a_i)/\phi + n_{ij} - y_{ij}\big) \\
-& \sum_j \log B\big(\sigma(-\boldsymbol{x}_{ij}^T\boldsymbol{\beta} + a_i)/\phi, \sigma(-\boldsymbol{x}_{ij}^T\boldsymbol{\beta} + a_i)/\phi\big)\Big] \\
=& \text{const} + \pi_i f_{\boldsymbol{\beta},\sigma_a^2,\phi,\boldsymbol{X}_i,\boldsymbol{y}_i,\boldsymbol{n}_i}(a_i) + (1-\pi_i) f_{-\boldsymbol{\beta},\sigma_a^2,\phi,\boldsymbol{X}_i,\boldsymbol{y}_i,\boldsymbol{n}_i}(a_i)
\end{aligned}$$

Here we dropped the subscript (t) for simpler notations. The derivatives can be computed as follows:

$$\begin{aligned}
h'(a_i) &= \pi_i f'_{\boldsymbol{\beta},\sigma_a^2,\phi,\boldsymbol{X}_i,\boldsymbol{y}_i,\boldsymbol{n}_i}(a_i) + (1-\pi_i) f'_{-\boldsymbol{\beta},\sigma_a^2,\phi,\boldsymbol{X}_i,\boldsymbol{y}_i,\boldsymbol{n}_i}(a_i) \\
h''(a_i) &= \pi_i f''_{\boldsymbol{\beta},\sigma_a^2,\phi,\boldsymbol{X}_i,\boldsymbol{y}_i,\boldsymbol{n}_i}(a_i) + (1-\pi_i) f''_{-\boldsymbol{\beta},\sigma_a^2,\phi,\boldsymbol{X}_i,\boldsymbol{y}_i,\boldsymbol{n}_i}(a_i)
\end{aligned}$$

Similar to section 3.1, we derive the maximum $\hat{a}_i$ using Newton-Raphson updates

$$a_i^{k+1} = a_i^k - \tau h'(a_i^k)/h''(a_i^k)$$

and $q(a_i)$ can be approximated by $N(\hat{a}_i, \hat{\sigma}_{a_i}^2)$, where $\hat{\sigma}_{a_i}^2 = |h''(\hat{a}_i)|^{-1}$. We choose $\tau = 0.9$.

4 Simulate haplotype proportions

In the simulation studies, we vary the LD coefficient (r^2) between the eQTL and the tSNP. The simulation model, however, does not directly use the LD coefficient. Instead, it uses haplotype proportions determined by r^2 and the minor allele frequencies (MAF).

We start by introducing a few notations. Denote by g_{r1} the genotype of eQTL (regulatory SNP) in haplotype 1 and g_{r2} the genotype of eQTL in haplotype 2. Similarly define g_{e1} and g_{e2} as the genotypes of tSNP in haplotypes 1 and 2, respectively. Genotypes $g_{r2}, g_{r2}, g_{e1}, g_{e2}$ can takes values 0 or 1. Denote by a_r and a_e the minor allele frequencies (MAF) of the eQTL and tSNP, respectively. We start by simulating a_r from a uniform distribution: $a_r \sim U[0.1, 0.5]$.

With a given r^2, the possible values of a_e are bounded by a_r. We derive the bound by first deriving the relationship among r^2, MAFs and haplotype frequencies. Note that

$$\begin{aligned} r =& \frac{E(g_{r1} + g_{r2} - Eg_{r1} - Eg_{r2})(g_{e1} + g_{e2} - Eg_{e1} - Eg_{e2})}{\sqrt{var(g_{r1} + g_{r2})var(g_{e1} + g_{e2})}} \\ =& \frac{E(g_{r1} + g_{r2} - 2a_r)(g_{e1} + g_{e2} - 2a_e)}{\sqrt{4a_r a_e(1 - a_r)(1 - a_e)}} \\ =& \frac{E(g_{r1}g_{e1}) + E(g_{r2}g_{e2}) - 2a_r a_e}{\sqrt{4a_r a_e(1 - a_r)(1 - a_e)}} \\ =& \frac{P(g_{r1} = 1, g_{e1} = 1) - a_r a_e}{\sqrt{a_r a_e(1 - a_r)(1 - a_e)}} \end{aligned}$$

Without loss of generality we assume $r > 0$. If $r < 0$ we can simply flip the reference and alternative alleles of one of the SNPs to ensure $r > 0$. Define the following haplotype frequencies:

$$\begin{aligned} p_{11} &= P(g_{r1} = 1, g_{e1} = 1), \quad p_{10} = P(g_{r1} = 1, g_{e1} = 0) \\ p_{01} &= P(g_{r1} = 0, g_{e1} = 1), \quad p_{00} = P(g_{r1} = 0, g_{e1} = 0) \end{aligned}$$

Hence $p_{11} = a_r a_e + r\sqrt{a_r a_e(1 - a_r)(1 - a_e)}$. It needs to satisfy the restrictions $p_{11} < a_r$ and $p_{11} < a_e$ since the haplotype frequency cannot exceed corresponding allele frequencies of individual SNPs. This is equivalent to

$$r^2 \frac{a_e}{1 - a_e} \leq \frac{a_r}{1 - a_r}, \quad r^2 \frac{a_r}{1 - a_r} \leq \frac{a_e}{1 - a_e}.$$

Hence we can derive the bounds for a_e:

$$\frac{r^2 a_r}{1 - a_r + r^2 a_r} \leq a_e \leq \frac{a_r}{r^2(1 - a_r) + a_r}.$$

We simulate a_e by uniform distribution: $a_e \sim U[\frac{r^2 a_r}{1-a_r+r^2 a_r}, \frac{a_r}{r^2(1-a_r)+a_r}]$. Finally, we calculate the haplotype frequencies by

$$\begin{aligned} p_{11} =& a_r a_e + r\sqrt{a_r a_e(1 - a_r)(1 - a_e)} \\ p_{01} =& a_e - p_{11}, \; p_{10} = a_r - p_{11}, \; p_{00} = 1 - p_{11} - p_{01} - p_{10}. \end{aligned}$$

Hence the mixture probabilities are calculated by

$$\tilde{\pi}_1 = 2p_{00}p_{11},\ \tilde{\pi}_2 = 2p_{01}p_{10},\ \tilde{\pi}_3 = 2p_{10}p_{11} + 2p_{00}p_{01}.$$

These are the proportions of individuals for which the eQTL is heterozygous ($\tilde{\pi}_1, \tilde{\pi}_2$) or homozygous ($\tilde{\pi}_3$) in the general population, regardless of whether the tSNP is heterozygous. However, we need to restrict to the individuals for which the tSNP is heterozygous, since ASE cannot be measured for homozygous individuals. Hence we normalize the probabilities to get the final mixture probabilities:

$$\pi_k = \frac{\tilde{\pi}_k}{\tilde{\pi}_1 + \tilde{\pi}_2 + \tilde{\pi}_3}, \quad k = 1, 2, 3.$$

5 References

1. Wang, Chong, and David M. Blei. "Variational inference in nonconjugate models." arXiv preprint arXiv:1209.4360 (2012).

2. Blei, David M., Alp Kucukelbir, and Jon D. McAuliffe. "Variational inference: A review for statisticians." Journal of the American statistical Association 112.518 (2017): 859-877.
