# Supplementary material for: Single-cell allele-specific expression analysis reveals dynamic and cell-type-specific regulatory effects
Source: Nat Commun. 2023 Oct 9;14:6317. doi: 10.1038/s41467-023-42016-9 (PMC10562474; doi:10.1038/s41467-023-42016-9)
Supplement: Supplementary file 3 — Description of Additional Supplementary Files [file 41467_2023_42016_MOESM3_ESM.pdf]

Description of Additional Supplementary Files

File Name: Supplementary Data 1
Description: Results from differential ASE analysis of endoderm differentiation data.

File Name: Supplementary Data 2
Description: GO biological process gene sets enriched in dynamic ASE genes by DAESC-Mix. Two pathways related to development are highlighted in bold.

File Name: Supplementary Data 3
Description: Number of significant genes (FDR<0.05) with or without adjusting for batch variables for D-ASE detection in the endoderm differentiation data

File Name: Supplementary Data 4
Description: Candidate coefficients for simulation studies.
