# Supplementary material for: Single-cell allele-specific expression analysis reveals dynamic and cell-type-specific regulatory effects
Source: Nat Commun. 2023 Oct 9;14:6317. doi: 10.1038/s41467-023-42016-9 (PMC10562474; doi:10.1038/s41467-023-42016-9)
Supplement: Supplementary file 5 — Reporting Summary [file 41467_2023_42016_MOESM5_ESM.pdf]

nature portfolio

Corresponding author(s): Alexis Battle

Last updated by author(s): Sep 8, 2023

Reporting Summary

Nature Portfolio wishes to improve the reproducibility of the work that we publish. This form provides structure for consistency and transparency in reporting. For further information on Nature Portfolio policies, see our Editorial Policies and the Editorial Policy Checklist.

Statistics

For all statistical analyses, confirm that the following items are present in the figure legend, table legend, main text, or Methods section.

n/a	Confirmed	
☐	☒	The exact sample size (*n*) for each experimental group/condition, given as a discrete number and unit of measurement
☐	☒	A statement on whether measurements were taken from distinct samples or whether the same sample was measured repeatedly
☐	☒	The statistical test(s) used AND whether they are one- or two-sided<br>*Only common tests should be described solely by name; describe more complex techniques in the Methods section.*
☐	☒	A description of all covariates tested
☐	☒	A description of any assumptions or corrections, such as tests of normality and adjustment for multiple comparisons
☐	☒	A full description of the statistical parameters including central tendency (e.g. means) or other basic estimates (e.g. regression coefficient) AND variation (e.g. standard deviation) or associated estimates of uncertainty (e.g. confidence intervals)
☐	☒	For null hypothesis testing, the test statistic (e.g. *F*, *t*, *r*) with confidence intervals, effect sizes, degrees of freedom and *P* value noted<br>*Give P values as exact values whenever suitable.*
☒	☐	For Bayesian analysis, information on the choice of priors and Markov chain Monte Carlo settings
☒	☐	For hierarchical and complex designs, identification of the appropriate level for tests and full reporting of outcomes
☐	☒	Estimates of effect sizes (e.g. Cohen's *d*, Pearson's *r*), indicating how they were calculated

Our web collection on statistics for biologists contains articles on many of the points above.

Software and code

Policy information about availability of computer code

Data collection	R/4.0.2
Data analysis	DAESC/0.1.0 (URL: https://github.com/gqi/DAESC)<br>R/4.0.2<br>Trimmomatic/v0.38<br>STAR/2.7.10a<br>Picard/2.18<br>GATK/4.0.0

For manuscripts utilizing custom algorithms or software that are central to the research but not yet described in published literature, software must be made available to editors and reviewers. We strongly encourage code deposition in a community repository (e.g. GitHub). See the Nature Portfolio guidelines for submitting code & software for further information.

Data

Policy information about availability of data

All manuscripts must include a data availability statement. This statement should provide the following information, where applicable:

- Accession codes, unique identifiers, or web links for publicly available datasets
- A description of any restrictions on data availability
- For clinical datasets or third party data, please ensure that the statement adheres to our policy

The ASE data, cell meta data, and gene expression from endoderm differentiation are available on https://zenodo.org/record/3625024#.YnJ-ivPMKi4. HipSci genotype data used in this study are available via: https://www.hipsci.org/lines/#/files?Assay%5B%5D=Genotyping%20array. The pancreatic islet data are available on ArrayExpress via accession number E-MTAB-5061 [https://www.ebi.ac.uk/biostudies/arrayexpress/studies/E-MTAB-5061]. GENCODE hg19 reference genome is available via: https://www.gencodegenes.org/human/release_44lift37.html. Source data are provided with this paper.

Human research participants

Policy information about studies involving human research participants and Sex and Gender in Research.

Reporting on sex and gender	The findings apply to both sexes. The donors for the endoderm differentiation dataset are 59.6% female and 40.4% male. The donors for the type 2 diabetes dataset consist of 3 female donors and 7 male donors. All data are publicly available and not consent was needed.
Population characteristics	Donors for the endoderm differentiation dataset are aged 25-79, with 68% donors aged 50-69; all donors are healthy. Donors for the type 2 diabetes data are aged 22-57, including 4 type 2 diabetes patients and 6 healthy controls.
Recruitment	No recruitment is needed for this study.
Ethics oversight	This study uses publicly available data. The approval of study protocol were obtained by the previous studies that collected the data.

Note that full information on the approval of the study protocol must also be provided in the manuscript.

Field-specific reporting

Please select the one below that is the best fit for your research. If you are not sure, read the appropriate sections before making your selection.

☒ Life sciences ☐ Behavioural & social sciences ☐ Ecological, evolutionary & environmental sciences

For a reference copy of the document with all sections, see nature.com/documents/nr-reporting-summary-flat.pdf

Life sciences study design

All studies must disclose on these points even when the disclosure is negative.

Sample size	The study uses publicly available data hence no sample size calculation was performed. The endoderm differentiation dataset includes 105 individuals; the pancreatic islet dataset includes 10 individuals.
Data exclusions	For the endoderm differentiation datasets, genes with allele-specific counts in <20% cells were excluded. For the type 2 diabetes dataset, we first remove individuals with <3 cells or <5 reads from the cell type. We drop the gene from D-ASE analysis if there are <50 cells or <2 cases or <2 controls remaining. These exclusions are imposed to ensure there are enough cells for differential ASE analysis.
Replication	The study focuses on development of statistical methods and uses publicly available data. For the endoderm differentiation data, we checked the overlap between our dynamic ASE genes and eGenes reported by Cuomo et al as an indirect measure of replication. Despite the difference in analysis methods, we observed 27-36% overlap between the two sets. No replication was conducted for the pancreatic islet data.
Randomization	No randomization was needed. Both datasets we used are publicly available and collected from observational studies. Our statistical models inherently adjusts for donor ID as covariates. We verified that additional batch effects had minimal effects in our analysis and no additional covariates were adjusted.
Blinding	The study only uses existing publicly available data from observational studies. No new experiments were conducted in this study, hence blinding was not applicable.

Reporting for specific materials, systems and methods

We require information from authors about some types of materials, experimental systems and methods used in many studies. Here, indicate whether each material, system or method listed is relevant to your study. If you are not sure if a list item applies to your research, read the appropriate section before selecting a response.

Materials & experimental systems

n/a	Involved in the study
☒	☐ Antibodies
☒	☐ Eukaryotic cell lines
☒	☐ Palaeontology and archaeology
☒	☐ Animals and other organisms
☒	☐ Clinical data
☒	☐ Dual use research of concern

Methods

n/a	Involved in the study
☒	☐ ChIP-seq
☒	☐ Flow cytometry
☒	☐ MRI-based neuroimaging
